# Distal ureter and bladder cuff excision using the “Keyhole Technique” during Robotic Radical Nephroureterectomy

**DOI:** 10.1590/S1677-5538.IBJU.2022.0147

**Published:** 2022-04-10

**Authors:** Luis G. Medina, Muhannad Alsyouf, Alireza Ghoreifi, Aref S. Sayegh, Kailyn Koh, Wenhao Yu, Sina Sobhani, Antoin Douglawi, Hooman Djaladat

**Affiliations:** 1 University of Southern California Norris Comprehensive Cancer Center Institute of Urology Los Angeles CA USA Institute of Urology, Norris Comprehensive Cancer Center, University of Southern California, Los Angeles, CA, USA

## Abstract

**Introduction:**

Upper tract urothelial carcinoma (UTUC) accounts for 5-10% of all urothelial tumors (1). Radical nephroureterectomy (RNU) remains the standard treatment for high, and low-grade UTUC (2). Although the open approach has been considered the gold standard, robotic techniques have shown comparable oncological outcomes with potential advantages in terms of peri-operative morbidity (3).

**Materials and Methods:**

We present a novel “Keyhole” technique for management of distal ureter and bladder cuff during robotic RNU. This technique allows the surgeon to directly visualize the ureteric orifices, delineate resection borders, and maintain oncologic principles of en-bloc excision without necessitating secondary cystotomy incision or concomitant endoscopic procedure. Descriptive demographic characteristics, surgical, pathological, and oncological outcomes were analyzed. Complications were reported using the Clavien-Dindo classification system.

**Results:**

Between 2015 and 2020, ten patients underwent robotic RNU with bladder cuff excision using the Keyhole technique (single-dock, single-position). Median age was 75 years. Eight patients underwent surgery for right-sided tumors. Median operative time, estimated blood loss, and length of hospital stay were 287 min, 100 mL, and 3 days, respectively. No intraoperative complications occurred, and one grade II complication occurred during the 90-day postoperative period. All patients had high-grade UTUC, being 90% pure urothelial. Bladder recurrences occurred in 30% of patients with an overall median follow-up of 11.2 months.

**Conclusions:**

Keyhole technique for the management of distal ureter and bladder cuff during RNU represents a feasible approach with minimal 90-day complications and low bladder recurrence rate at centers of experience.

## References

[1] Siegel RL, Miller KD, Jemal A. Cancer statistics, 2019. CA Cancer J Clin. 2019;69:7-34. 10.3322/caac.21551 30620402

[2] Rouprêt M, Babjuk M, Burger M, Capoun O, Cohen D, Compérat EM, et al. European Association of Urology Guidelines on Upper Urinary Tract Urothelial Carcinoma: 2020 Update. Eur Urol. 2021;79:62-79. 10.1016/j.eururo.2020.05.042 32593530

[3] Piszczek R, Nowak Ł, Krajewski W, Chorbinska J, Poletajew S, Moschini M, et al. Oncological outcomes of laparoscopic versus open nephroureterectomy for the treatment of upper tract urothelial carcinoma: an updated meta-analysis. World J Surg Oncol. 2021;19:129. 10.1186/s12957-021-02236-z PMC8061074 33882936

